# Transcription factor Sp1 induces ADAM17 and contributes to tumor cell invasiveness under hypoxia

**DOI:** 10.1186/1756-9966-28-129

**Published:** 2009-09-22

**Authors:** Alexandra Szalad, Mark Katakowski, Xuguang Zheng, Feng Jiang, Michael Chopp

**Affiliations:** 1 Department of Neurology, Henry Ford Hospital, Detroit, Michigan 48202, USA; 2 Physics Department, Oakland University, Rochester, Michigan 48309, USA

## Abstract

**Background:**

Expression of the Sp1 transcription factor is induced by hypoxia, and the ADAM17 promoter contains predicted Sp1 binding sites. ADAM17 contributes to hypoxic-induce invasiveness of glioma. In this study, we investigated whether Sp1 transcription factor induces ADAM17 and/or contributes to tumor cell invasiveness in hypoxia.

**Methods:**

Employing RT-PCR and Western blot, we examined the role of Sp1 in ADAM17 transcription/expression under normoxic and hypoxic conditions, and whether it binds to the ADAM17 GC-rich promoter region using a chromatin immunoprecipitation assay. Additionally, we tested the effect of Sp1 suppression in tumor cell invasion and migration, using Matrigel basement membrane invasion chambers, a scratch wound-healing assay, and small interfering RNA.

**Results:**

Here, we found that Sp1 binds to the ADAM17 promoter, and that Sp1 regulates ADAM17 expression under hypoxia. Furthermore, suppression of Sp1 decreases invasiveness and migration in U87 tumor cells.

**Conclusion:**

Our findings suggest the Sp1 transcription factor mediates ADAM17 expression under hypoxia, regulates glioma invasiveness, and thus, may be a target for anti-invasion therapies.

## Background

Brain tumors result from abnormal or uncontrolled cell division. Glioma is a highly vascular, very aggressive and extremely invasive primary brain tumor. Hypoxia induces changes in glioma and its microenvironment, which leads to increased aggressiveness and resistance to chemotherapy and radiation [1]. Studies have shown that large areas of hypoxia within glioma correlates inversely with the patient's outcome and survival [1-4].

ADAM17 (A Disintegrin and Metalloproteinase-17) also called TACE (TNF-alpha converting enzyme) plays a pivotal role in the processing of numerous growth factor proteins, and has emerged as a new therapeutic target in several tumor types [5-8]. Recent studies showed that when ADAM17 is either inhibited or suppressed there is attenuation in tumor invasiveness and malignancy, resulting in a better outcome for breast cancer patients [9,10].

Low levels of oxygen (hypoxia) initiates cellular invasive processes that occur under physiological and pathological conditions such as tumor invasiveness and metastasis [11].

Specificity transcription protein-1 (Sp1) is believed to play an important role in the transcription of many genes involved in cancer that have an abundance of GC boxes in their promoter region [12-15]. Currently, the role of Sp1 in ADAM17 expression and activity is unknown, but it is known the ADAM17 promoter region contains GC-rich sequences highly complementary to the Sp1 DNA-binding site [16]. Hypoxia induces expression of ADAM17 and increases invasiveness of glioma in vitro [6].

In this study, we investigated if Sp1 protein plays a role in ADAM17 transcription, and if Sp1 regulates hypoxic-induced ADAM17 expression in U87 human glioma cells. In addition, we examined the function of Sp1 in tumor invasiveness under normoxic and hypoxic conditions.

## Methods

### Cell culture

The U87 tumor cell line was obtain from American Type Culture Collection (ATCC) The cells were grown in DMEM (Dulbeco Modified Essential Medium) which contained 10% FBS (Fetal Bovine Serum),100 IU/mL penicillin, 100 μg/mL streptomycin (Life Technologies). The cells were passed once a week after trypsinization (0.05% trypsin-ethylenediaminetetraacetic acid; Life Technology).

### Hypoxic culture conditions

The hypoxia experiments were performed in an anaerobic chamber (model 1025; Forma Scientific) which was saturated with 85%N_2_/10%H_2_/5%CO_2_. The temperature in the anaerobic chamber was set at 37°C and the oxygen level was below 1%. The media was changed before the experiment with DMEM low glucose and 10% FBS. The cells were harvested at 8, 12, 16 and 20 hours. In parallel with the hypoxic culture, normoxic culture was harvested as well to serve as a control for all assays.

### Chromatin immunoprecipitation assay (ChIP)

ChIP assay was performed as follows: U87 (1 × 10^6^) cells on a 10 cm dish were treated with 1% formaldehyde and incubated for 10 min at 37°C, then washed with cold PBS containing protease inhibitors (1 mM phenylmethylsulfonyl fluoride-PMSF, 1 μg/mL aprotinin and 1 μg/mL pepstatin A). The cells were collected, spun down and added SDS lysis buffer, and then incubated on ice. The DNA was sonicated (5 pulses for 10 s, chilled on ice for 50 s) to shear it into 200-1000 base pairs. Once the sheared DNA was diluted into ChIP buffer a pellet was obtain by centrifugation. The assay requires two negative controls. The first control was transcriptionally inactivated DNA that was used for the PCR reaction, and the second control was transcriptionally active DNA without antibody for immuno-precipitation. The immuno-precipitating Sp1 antibody was added to the DNA and incubated overnight. PCR (Polymerase Chain Reaction) was done in order to amplify the DNA that was bound to the immunoprecipitated histones. The primers used for amplification were design using OligoPerfect Primer Design Program (Invitrogen) and are as follows: A17 1F 5'-TGGAGCAAATGTGCATTCAG-3', A17 1R 5'-GCATTTGGTTCAGGGTCCTA-3', A17 2F 5'- GTGGGCATCAAGACAAAGGA-3', A17 2R 5'-CTTCCTGGACGCAGACGTA-3', A17 3F 5'-GAGCCTGGCGGTAGAATCTT-3', A17 3R 5'-TACCGACTCCACCTCTCTGG-3'. Once amplified, the PCR product was tested by electrophoresis on a 2% agarose gel containing 0.01% ethidium bromide. The results were visualized using DualLite Trans-illuminator machine (Fisher). The ChIP assay was performed under normoxic conditions.

### Real-time PCR

Quantitative RT-PCR was performed using real-time PCR with the SYBR Green reporter. The RNA was isolated from the cell cultures by using the Absolutely RNA Miniprep Kit (Stratagene). RNA yield was determined with OD260 nm. RNA was reverse transcribed to complementary DNA using the M-MLV RT protocol (Invitrogen). Quantitative RT-PCR was performed after stabilizing the RNA. The kit used for RT-PCR was a SYBR Green PCR master kit with the appropriate forward and reverse primers (Invitrogen), which were optimized to the desired concentration (10 nM). The instrument used for this experiment was ABI 7000 PCR machine (Applied Biosystems). Each sample was tested three times. The primers used for this experiment are in Table 1. Human TATA-box binding protein was used as an internal control.

**Table 1:** The primers used for real time polymerase chain reaction

**Gene**	**GenBank accession number**	**Sequence**
HIF-1α	NM024359	5'-CGTTCCTTCGATCAGTTGTC -3'
		5'-TCAGTGGTGGCAGTGGTAGT -3'

ADAM17	NM003183	5'-ACTCTGAGGACAGTTAACCAAACC-3'
		5'-AGTAAAAGGAGCCAATACCACAAG-3'

Sp1	NM138473	5'-AAACATATCAAAGACCCACCAGAAT-3'
		5'-ATATTGGTGGTAATAAGGGCTGAA-3'

TBP	NM003194	5'-TGCACAGGAGCCAAGAGTGAA-3'
		5'-CACATCACAGCTCCCCACCA-3'

ADAM17, a disintegrin and a metalloproteinase-17; HIF-1α, hypoxia inducible factor-1 alpha; Sp1, specificity transcription protein -1; TBP, TATA-binding protein.

### Western blot

Proteins were extracted from the cell culture and the added in 500 μL lysis buffer with 1% protease inhibitor cocktail (1 mM phenylmethylsulfonyl fluoride-PMSF, 1 μg/mL aprotinin and 1 μg/mL pepstatin A). The concentration of the proteins was determined by Bicinchoninic acid assay (BCA) following the protocol provided by the manufacturer (Pierce). Equal amounts of proteins mixed with NuPage loading buffer were loaded on a 12% Bris-Tris Gels (Invitrogen) after being denatured. After blockage the membrane was incubated using primary antibodies, which were added against ADAM17 (Abcam), HIF-1α (Cell Signaling), Sp1 (Santa Cruz) or β-Actin (Abcam) in PBS-T containing 5% milk overnight at 4°C. Subsequently, the membrane was washed with PBS-T for 45 minutes at room temperature, followed by incubation with the secondary antibodies for 1 hr and 30 min, still at room temperature. The immunoblots were detected, following a second washing, using the SuperSignal West Pico Chemiluminescent Substrate kit (Pierce). The internal control for Western blots was β-Actin.

### Alpha-secretase assay

After U87 tumor cells were harvested, lysis buffer was added to the tubes. Proteins were sonicated five times for 10 sec each time. A BCA protein assay (Thermo Scientific) was done in order to find the protein concentration of each sample. A total volume of 200 μl proteins with buffers were added to the alpha-secretase specific APP (amyloid precursor protein) peptide. Two wells were used as control, where only buffer was added to them. Fluorescence was read using a Fusion multiplate reader (Packard Bioscience).

### In vitro invasion assay

Matrigel invasion assay (BD Biosciences) was employed to test cell invasion. The membrane was soaked in DMEM low glucose and incubated for one hour at 37°C. The bottom well contained high glucose DMEM containing serum. Cells were added to the upper well and incubated for 24 hours at 37°C with 5% CO_2_. After incubation, Cell Tracker (Invitrogen) dye was added to the wells for 20 minutes. Cells were fixed with 4% paraformaldehyde; the membrane was removed, and then transferred to slides for analysis.

### In vitro wound-repair assay

This assay was used to assess cell migration. In a 24-well plate, U87 tumor cells were added to high glucose DMEM media, and incubated for two hours in order to create a monolayer of cells. A scratch was made in the middle of the well with a p200 pipette tip. The debris was washed away and new media added to the wells. Under the microscope, the cells were imaged and the initial area of the scratch for the field of view was determined by multiplying the length by the average width of the area devoid of cells. The plate was incubated at 37°C for 12 hours, after which the same field of view was imaged and the area devoid of cells was recalculated by the same method. The final area of the scratch wound was divided by the initial area, giving us the % of the initial area covered by migrating cells over the 12 hours culture period.

### siRNA transfection

A stable transfection of Sp1 siRNA was carried out using Lipofectamine 2000 transfection reagent (Invitrogen). The transfection reagent and the siRNA were diluted in 100 μL DMEM without serum or antibiotic. Once they were mixed together, they were incubated for 20 minutes at room temperature. For each transfection 6 mL DMEM was added to each tube containing the siRNA-transfection mixture. Clonal selection of neomycin-resistant U87 cells was conducted after transfection. Sp1 down-regulation was verified in transfected U87 clones using Western blot. The cells were maintained in neomycin-containing media, and employed less than 10 passages after confirmation of reduced Sp1 protein expression. Of note, Sp1 down-regulation in U87 cells caused cells to acquire a flat, less bipolar morphology compared to control transfected cells. All Sp1 shRNA-expressing clones shared this morphology whereas control plasmid transfected clones did not, suggesting the effect was due to Sp1 down-regulation.

## Results and discussion

### Sp1 binds to the ADAM17 promoter

**Figure 1:**
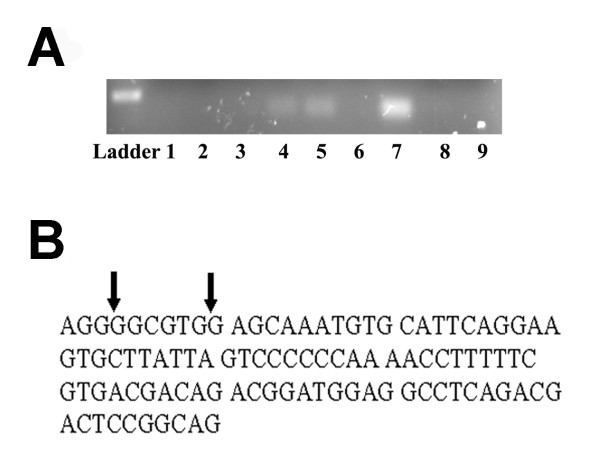
**A. Chromatin Immuno-Precipitation analysis of Sp1 binding to the ADAM17 promoter**. Lanes 1-3 are negative controls for immuno-precipitation. Lanes 4-6 are the negative controls for the DNA optimization. The band in lane 7 indicates Sp1 binding within the ADAM17 promoter within 1-97 bp sequence. Lanes 8 and 9 indicate no Sp1 binding for the 356-455 and 781-879 regions of the ADAM17 promoter, respectively. **B**. The promoter sequence of ADAM17 from base pair one up to base pair 97. The arrows indicate the predicted human Sp1 binding site (3-9 bp).

Sp1 binds to GC boxes in the promoter region of genes to regulate their expression. It has been suggested that ADAM17 is one of these genes [16]. Using the ChIP assay, we tested whether the Sp1 transcription factor binds to the ADAM17 promoter region. Employing three fragments of the ADAM17 promoter (GenBank: AB034151.1), results of PCR amplification indicated Sp1 bound to the fragment corresponding to the first 97 bp of the ADAM17 promoter region (Figure 1A), corresponding to (1-97 of AB034151.1, -901 to -804 of the ADAM17 initiation codon). The human Sp1 consensus sequence starts at base pair 3 and the length is 6 base pairs long, indicating a probable binding site (Figure 1B).

### Hypoxia up-regulates ADAM17 and Sp1 in U87 tumor cells

**Figure 2:**
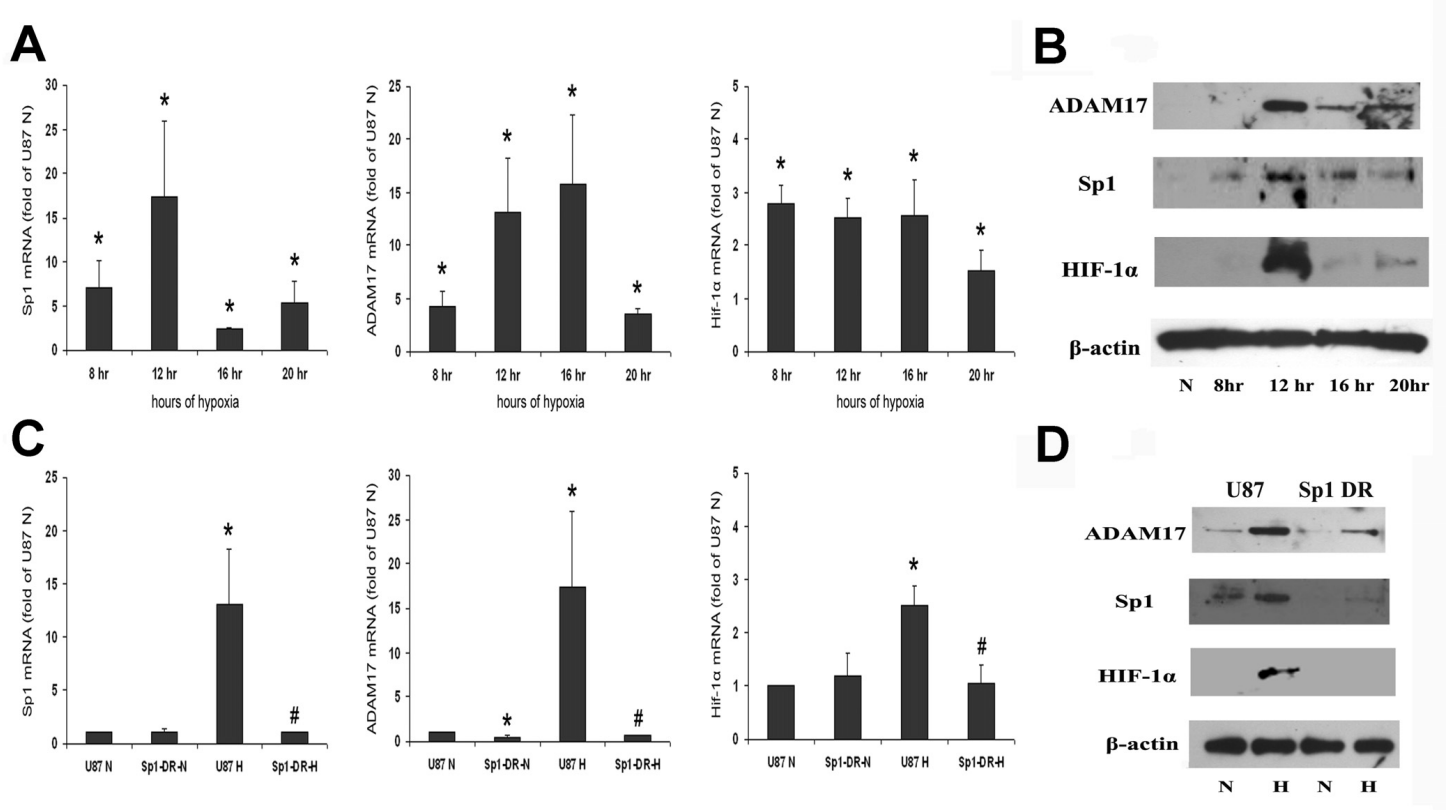
**Real-time RT-PCR and Western blot for Sp1, ADAM17 and HIF-1α in U87**. N: normoxic incubation, H: hypoxic incubation, the 8 thru 20 hours indicate time points of hypoxic incubation. Sp1-DR: stable U87 cells expressing Sp1 siRNA. **A**. RT-PCR of U87 cells subjected to normoxic and hypoxic incubation for 8, 12, 16 and 20 hours. ADAM17, Sp1 and HIF-1α mRNA levels significantly increase under hypoxic conditions, peaking at 12 hr incubation.*P < 0.05 compared to normoxic control. **B**. U87 cells harvested for Western blot were incubated under normoxic and hypoxic conditions. ADAM17, Sp1 and HIF-1α proteins increased under hypoxic conditions, peaking after 12 hr hypoxic incubation. **C**. RT-PCR after 12 hour hypoxic incubation of U87 control and Sp1-deficient U87 cells. Sp1 down-regulation significantly decreased mRNA levels of Sp1, ADAM17 and HIF-1 α. *P < 0.05 compared to normoxic control. #P < 0.05 compared to hypoxic control. **D**. Western blots after 12 hour hypoxic incubation of U87 control and Sp1-deficient U87 cells. Lanes 1 and 2: U87 control. Lanes 3 and 4: Sp1-deficient U87 cells. ADAM17, Sp1 and HIF-1α decreased compared to the control under hypoxic conditions.

Real-time RT-PCR was performed to determine whether Sp1 transcription factor mediates ADAM17 expression under normoxic and hypoxic conditions. Real-time RT-PCR analysis of ADAM17, Sp1 and HIF-1α mRNA was performed on U87 tumor cells. Human TATA-Box protein was used as a normalizing control, and HIF-1α was used as a positive marker for hypoxia. The mRNA samples used for PCR were normoxic control, 8 hours, 12 hours, 16 hours and 20 hours of hypoxia. Sp1 mRNA expression peaked after 12 hours of hypoxic incubation. Significant increases (*P < 0.05) were observed in the mRNA levels of ADAM17, Sp1 and Hif-1α genes under hypoxic compared to normoxic conditions (Figure 2A). To test the contribution of Sp1 to ADAM17 expression, we established a Sp1-deficient cell-line by transfecting U87 cells with a plasmid encoding for Sp1-targeting siRNA. U87 cells transfected with empty pcDNA3.1+ vector were used as control. Employing these cells in our hypoxic incubation assay, we found that the mRNA expression levels of ADAM17 and Sp1 were significantly (*P < 0.05) lower compared to the results obtained from respective control (Figure 2C). Of note, HIF-1α mRNA levels were also affected by inhibition of Sp1, and were significantly decreased compared to control HIF-1α mRNA expression under hypoxic conditions (Figure 2C). This is likely due to the fact that Sp1 is a known transcription factor for HIF-1α [17]. These results suggest that ADAM17 mRNA expression is altered by the Sp1 transcription factor, particularly ADAM17 transcription induced by hypoxia.

Western blot was employed to determine the protein expression of Sp1, ADAM17 and HIF-1α. In addition, we tested whether Sp1 down-regulation affects ADAM17 expression levels under normoxic and hypoxic conditions. β-Actin protein was used as a loading control and HIF-1α protein was used as a positive marker for hypoxia. Western blotting revealed an increase ADAM17, Sp1 and HIF-1α protein expression under hypoxic conditions compared to normoxic control. The blots of all three proteins increased under hypoxia, and peaked at 12 hours of hypoxic incubation within the time points where expression was measured (Fig 2B). When Sp1-deficient cells were used for the experiment, a significant decrease in ADAM17 protein expression levels was observed after 12 hours of culture, both under normoxic and hypoxic conditions (Figure 2D). These data indicate that under hypoxic conditions ADAM17 and Sp1 protein levels increased significantly but decreased when Sp1 is down-regulated. In addition, ADAM17 protein is decreased in Sp1 deficient cells under normoxic conditions as well. Based on these results, we concluded that the Sp1 transcription factor plays a significant role in controlling ADAM17 protein expression, particularly under hypoxic conditions. As Sp1 and ADAM17 protein expression peaked at 12 hours hypoxia, we employed this time point for our further hypoxic assays.

### Hypoxic-induced alpha-secretase assay in U87 is Sp1 dependent

**Figure 3:**
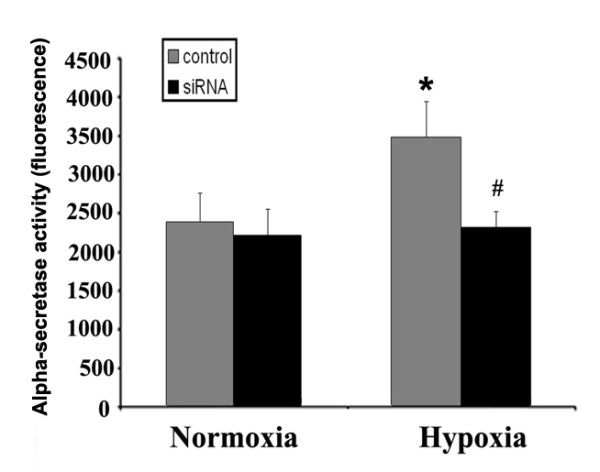
**Effect of Sp1 small interfering RNA (siRNA) on alpha-secretase activity in U87 tumor cells under normoxic and hypoxic conditions**. The incubation period was 12 hours. Alpha-secretase activity was significantly increased for U87 control cells under hypoxic compared to normoxic conditions. Sp1 suppression reduced alpha-secretase activity in hypoxic conditions. *P < 0.05 compared to normoxic control. #P < 0.05 compared to hypoxic control.

Previously, we reported that ADAM17 contributes to hypoxic-induced tumor invasion [6]. Having established that Sp1 mediates hypoxic-induced ADAM17 expression, we tested whether Sp1 down-regulation would elicit an anti-invasion effect, similar to inhibition of ADAM17. ADAM17 is an alpha-secretase, capable of proteolytic cleavage of APP into its soluble APP-alpha peptide [18]. Therefore we tested if the Sp1 transcription factor alters ADAM17 alpha-secretase activity in normoxic and hypoxic conditions. Hypoxic incubation of U87 for 12 hours increased alpha-secretase activity by 43.6% compared to normoxic control (Figure 3). This agreed with our previous findings that hypoxia induced alpha-secretase activity in U87 cells, primarily via ADAM17 [6]. In contrast, when Sp1 was suppressed, alpha-secretase activity under hypoxic incubation was unchanged compared to normoxic conditions (Figure 3). Notably, Sp1 suppression under normoxic conditions did not reduce alpha-secretase activity, suggesting Sp1 was critical for hypoxic-induced alpha-secretase activity, but not under normoxic conditions. These results suggest that Sp1 is a major contributor in hypoxic-induced alpha-secretase activity, possibly via suppression of hypoxia-induced ADAM17.

### Hypoxic-induced invasion and migration of U87 cells is Sp1 dependent

**Figure 4:**
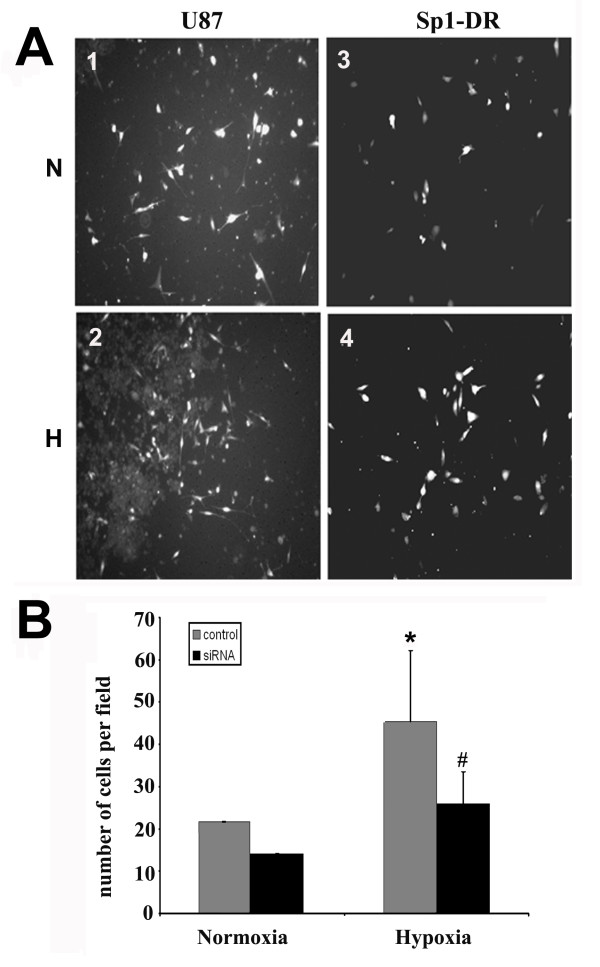
**Effect of Sp1 siRNA transfection upon invasiveness of U87 tumor cells under normoxic and hypoxic conditions**. **A**. U87 cell invasion at 4 × objectives: N: normoxic incubation, H: hypoxic incubation, U87: control cells, Sp1-DR: U87 cells expressing Sp1 siRNA. **1**. U87 control cells with transfected empty vector under normoxic conditions. **2**. U87 control cells subjected to hypoxic incubation. **3**. Sp1-deficient U87 cells under normoxic conditions. **4**. Sp1-deficient U87 cells under hypoxic conditions. **B**. Invasive cell number compared to normoxic control. *P < 0.05 compared to normoxic control. #P < 0.05 compared to hypoxic control.

Recently, we reported that the increased invasion ability of U87 cells is mediated by elevated ADAM17 expression and protease activity, particularly under hypoxic conditions [6,19]. In this assay we investigated whether Sp1 down-regulation elicits the same anti-invasion effect as inhibition of ADAM17 on tumor cells under hypoxia. An *in vitro *Matrigel invasion assay revealed that the invasiveness of U87 cells incubated in 1% oxygen was 52% higher compared to invasion under normoxic control conditions (Figure 4A). Furthermore, Sp1 suppression reduced the invasiveness of U87 cells by 17.3% in normoxic conditions and by 28.9% under hypoxic conditions compared to U87 control cells (Figure 4B). These results indicate the Sp1 transcription factor contributes to the invasive phenotype of U87 tumor cells.

**Figure 5:**
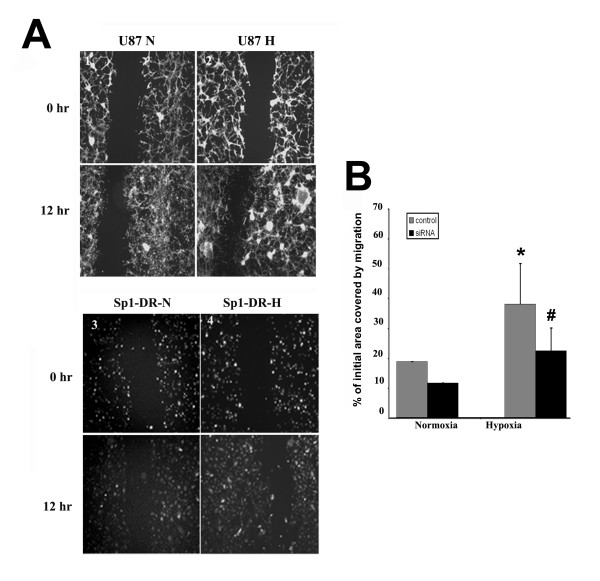
**Effect of Sp1 suppression upon migration of U87 tumor cells under normoxic and hypoxic conditions**. **A**. U87 cell migration at 4× objective. N: normoxic incubation, H: hypoxic incubation, 0 hr: zero hour incubation period, 12 hr: twelve hours incubation period, U87: control cells, Sp1-DR: U87 cells expressing Sp1 siRNA. **1**. U87 control cells under normoxic conditions. **2**. U87 control cells under hypoxic incubation. **3**. U87 cells expressing Sp1 siRNA under normoxic conditions. **4**. U87 cells expressing Sp1 siRNA under hypoxic conditions. **B**. Data are shown as percentage of the initial area covered by migration. *P < 0.05 compared to normoxic control. #P < 0.05 compared to hypoxic control.

Here, we established that the Sp1 transcription factor regulates ADAM17 expression under hypoxic conditions. As ADAM17 increases glioma invasiveness, we investigated whether Sp1 has functional consequence in glioma cell migration. To this end, we employed the *in vitro *scratch wound-repair assay to assess the migration ability of U87 and Sp1-deficient U87 cells under hypoxic conditions. The assay revealed that U87 tumor cells migrated 67.5% faster under hypoxic conditions than under normoxic conditions (Figure 5A). In contrast, Sp1 suppression decreased migration of U87 cells under both normoxic and hypoxic conditions (Figure 5B), and Sp1-deficient cell migrated 34.5% slower under hypoxic conditions compared to U87 controls.

## Concluding remarks

Current literature provides evidence of an association between hypoxic conditions and the difficulties of treating brain tumors, like glioma. Hypoxia has been implicated in many aspects of tumor development, angiogenesis and growth [2]. At the cellular level, hypoxia induces the expression and cellular concentration of HIF-1α. High expression of this factor leads to an increase in cell division-tumorigenesis and appears to be a prognostic marker for malignancy [19,20].

ADAMs comprise a family of proteins that contain both a disintegrin and a Zn-dependent metalloproteinase [21]. These molecules are involved in gene regulation, cell adhesion and proteolysis. The most extensively studied protein belonging to this family is ADAM17 (a.k.a. TACE). ADAM17 sheds a variety of epidermal growth factors receptor (EGFR)-binding ligands, including transforming growth factor-alpha (TGF-α), heparin-binding epidermal growth factor (HB-EGF), and amphiregulin [6,22]. In addition, ADAM17 facilitates the release of several integrins from the cell surface, and influences the invasive activity of several cells, including brain tumor [6,21]. Sp1 is important to the transcription of many genes that contain GC boxes in their promoters [23]. Sp1 has been widely perceived as a basal transcription factor since its discovery; however, increasing evidence suggests Sp1 regulates a multiple functions critical to tumorigenesis and progression [12,14,23]. Knowing that ADAM17 contributes to the invasiveness of tumor cells and that Sp1 binds to its promoter region, it is possible that Sp1 transcription factor may be a new target for anti-invasive therapies [14,23].

Previously, we have reported that the increased invasion ability of U87 cells under hypoxic conditions is mediated by elevated ADAM17 expression and protease activity [6,19]. Sp1 protein expression has been reported to increase in tumor cells under hypoxic conditions [24]. We used the TESS promoter analysis program to determine if the Sp1 transcription factor binds to ADAM17, as the promoter region of ADAM17 contained multiple Sp1 transcription factor binding sites [16]. Using a DNA-protein binding assay under normoxic conditions we found that Sp1 binds to ADAM17 within the ADAM17 promoter region, -901 to -804 of TSS. As one consensus sequence for human Sp1 is found at bp 3-9 of the ADAM17 promoter, we surmise this is the position of Sp1-binding; however mutational analysis is needed to confirm this is the target site. Sp1 down-regulation reduced expression of ADAM17 under both normal and hypoxic conditions; however, we have not confirmed the Sp1 binding site within the ADAM17 promoter is functional. Furthermore, it has been demonstrated that hypoxia can not only alter expression, but enhance the binding activity of Sp1 [24]. Thus, although we demonstrate binding of Sp1 to the ADAM17 promoter, further investigation of its transcriptional effect upon ADAM17 is warranted.

Previous studies have shown that at the transcriptional level, Sp1 plays a critical role in gene expression especially under hypoxic conditions [12,23,25]. Our PCR data revealed that hypoxia induced mRNA expression of ADAM17 as well as Sp1. In addition, we observed that our Sp1-deficient cells decreased mRNA expression of ADAM17 under both normoxic and hypoxic conditions. Using Western blot, we confirmed that hypoxia induced protein expression of ADAM17 and Sp1. However, when Sp1 was down-regulated by an expression plasmid encoding for siRNA, hypoxia failed to induce ADAM17 mRNA and protein expression indicating that Sp1 is required for hypoxic-induction of ADAM17.

Previously, we have reported that increased ADAM17 expression and protease activity contributes to hypoxic-induced tumor invasion. In this study, we established that Sp1 regulates ADAM17 gene expression. Furthermore, we investigated whether inhibition of Sp1 would elicit an anti-invasion effect similar to inhibition of ADAM17. Here, we used an alpha-secretase assay to determine if Sp1 siRNA influences ADAM17 protease activity. As expected, hypoxia increased alpha-secretase activity in U87 tumor cells. However, when Sp1 was down-regulated, hypoxia did not significantly increase alpha-secretase activity in line with inhibition of hypoxia-induce ADAM17. Of note, Sp1 down-regulation did not decrease alpha-secretase activity under normoxic conditions. This is in agreement with our previous data that ADAM17 does not constitute for the majority of alpha-secretase activity in U87 cells under normoxic conditions, but does account for the majority of hypoxic-induced alpha-secretase activity [6].

ADAM17 mediates hypoxic-induced glioma invasion [5,6,26]. To test if Sp1 contributes to the invasion of tumor cells, we used an *in vitro *invasion assay. Our results indicate that under hypoxic conditions the invasive ability of U87 significantly increased, and this increase was correlated with high ADAM17 expression and proteolytic activity. The invasive ability of U87 cells decreased considerably when Sp1 was suppressed under both normoxic and hypoxic conditions. Similar to invasion, Sp1 down-regulation resulted in a significant reduction in U87 cell migration both under hypoxic and normoxic conditions.

Here we demonstrate that Sp1 is critical for hypoxic-induced ADAM17, and that Sp1 contributes to hypoxic induced glioma invasion. However, we have not established the effect of Sp1 upon invasion is solely mediated via ADAM17. In addition to many other genes, HIF-1α contains Sp1 binding sites in its promoter [17]. In fact, we found Sp1 down-regulation diminished HIF-1α expression. Furthermore, the inhibitory effects of Sp1 down-regulation upon cell invasion and migration were more pronounced under hypoxic conditions, suggesting the role of Sp1 is more pronounced in the context of hypoxic-inducible factors. Hypoxic-induced ADAM17 expression is dependent upon Sp1, and ADAM17 significantly contributes to hypoxic-induced glioma invasion [6]. However, it is probable the effect of Sp1 upon hypoxic-induced cell invasion includes factors in addition to ADAM17.

Our study suggests that Sp1 transcription factor mediates hypoxia-induced ADAM17 expression and proteolytic activity, and contributes to an increase in invasiveness of brain tumor cells under normoxic and hypoxic conditions. These findings suggest that Sp1 may be a novel target for anti-invasive therapies of brain tumor.

## Competing interests

The authors declare that they have no competing interests.

## Authors' contributions

In our study all authors are in agreement with the content of the manuscript. Each author's contribution to the manuscript: AS: First author, study design, experimental studies, data analysis, manuscript editing. MK: study design, data analysis, manuscript editing. XZ: study design, setting up the siRNA cell line. FJ: study design and coordination, manuscript preparation. MC: Correspondent author study design and coordination, manuscript preparation. All authors read and approved the final manuscript.
